# SSB: Smart Contract Security Detection Tool Suitable for Industrial Control Scenarios

**DOI:** 10.3390/s25154695

**Published:** 2025-07-30

**Authors:** Ci Tao, Shuai He, Xingqiu Shen

**Affiliations:** College of Computer Science and Artificial Intelligence, Fudan University, Shanghai 200437, China; 24210240304@m.fudan.edu.cn (C.T.); 22210240162@m.fudan.edu.cn (S.H.)

**Keywords:** industrial control systems, smart contracts, blockchain security, vulnerability detection

## Abstract

The results of this study highlight the effectiveness of the proposed semantic security detection framework, SSB, in identifying a wide range of vulnerabilities in smart contracts tailored for industrial control scenarios. Compared to existing tools like ZEUS, Securify, and VULTRON, SSB demonstrates superior logical coverage across various vulnerability types, as evidenced by its performance on smart contract samples. This suggests that semantic-based approaches, which integrate domain-specific invariants and runtime monitoring, can address the unique challenges of ICS, such as real-time constraints and semantic consistency between code and physical control logic. The framework’s ability to model industrial invariants—covering security, functionality, consistency, time-related, and resource consumption aspects—provides a robust mechanism to prevent critical errors like unauthorized access or premature equipment operation. However, the lack of real-world ICS validation due to confidentiality constraints limits the generalizability of these findings. Future research should focus on adapting SSB for real industrial deployments, exploring scalability across diverse ICS architectures, and integrating advanced AI techniques for dynamic invariant adjustment. Additionally, addressing cross-chain interoperability and privacy concerns could further enhance the framework’s applicability in complex industrial ecosystems.

## 1. Introduction

In the process of intelligent transformation of industrial control systems (ICS), traditional security mechanisms struggle to address threats from open networks [1,2]. Although blockchain smart contracts hold application potential [3,4,5,6], their semantic security in industrial control scenarios faces significant challenges: the discrepancy between the physical semantics of control logic and the execution semantics of smart contracts may lead to security issues [7,8,9].

With the rapid development of Industry 4.0 and the Industrial Internet of Things (IIoT) [10,11,12], traditional ICS, which rely on technologies like PLCs and are secured by physical isolation, are becoming vulnerable to complex cyber-attacks in open networks [2]. Blockchain technology offers a novel approach to enhance ICS security through its decentralized, immutable, and traceable nature [3,13,14,15]. Smart contracts, in particular, enable the automatic execution of predefined logic, showing great potential for industrial automation [16,17]. However, the security of these contracts in the demanding ICS environment remains a critical concern [7,18,19].

Current smart contract security analysis tools can be broadly categorized as shown in Table 1. Static analysis tools like Slither scan source code for known vulnerability patterns [20], while dynamic analysis tools like Mythril execute contracts to find bugs [21]. More rigorous formal verification methods, exemplified by the Move Prover and tools using the K-framework, can mathematically prove contract correctness against a specification but often require significant manual effort [22,23,24,25]. Recently, large language models (LLMs) have been employed to detect a wider range of vulnerabilities by understanding code semantics [26,27,28].

Despite this progress, a significant gap remains. Existing tools are largely designed for financial applications on public blockchains [35,36] and often fail to address the unique challenges of ICS environments [1,37]. These challenges include stringent real-time constraints, the need for semantic consistency between code and physical control logic, and domain-specific safety properties [10]. A reentrancy bug in a DeFi contract may lead to financial loss [8,38], but a semantically incorrect state transition in an ICS contract could cause physical equipment damage or production shutdown [5]. This issue of **“Semantic Security”**—ensuring the contract’s execution aligns with the physical process logic—is not adequately addressed by existing methods [16,18,25].

To address the aforementioned challenges, this paper presents SSB, a semantic security detection framework for smart contracts tailored to industrial control scenarios. The main contributions are:

- By constructing an ontology model for the industrial control domain, we establish semantic mapping invariants from physical control requirements to smart contract code, resolving the challenge of semantic consistency.
- By integrating runtime monitoring and invariant detection techniques, we propose a semantic security detection framework (SSB) tailored to industrial control scenarios.

This research aims to provide theoretical and practical support for the secure deployment of smart contracts in industrial control systems, promoting the deep integration of blockchain technology with industrial automation.

## 2. Related Work

The secure application of smart contracts, especially in critical domains like ICS, requires robust analysis techniques. This section reviews existing approaches and situates our work within the current research landscape.

Early efforts in smart contract security focused on syntactic vulnerability detection, with seminal work highlighting common pitfalls like reentrancy [20]. Tools like ZEUS [39] and Securify [33] pioneered the field. ZEUS uses policy-based verification to check for safety properties but has limited coverage for other vulnerability types. Securify decompiles EVM bytecode to infer semantic facts and matches them against compliance and violation patterns. While powerful, its effectiveness is bound by its predefined pattern library and it lacks focus on domain-specific logic, such as the temporal constraints critical in ICS. VULTRON [34] introduced a novel approach based on balance invariants, monitoring global balance changes to detect anomalous financial transactions. However, this financial-centric model is not directly applicable to the non-financial state and logic validation required in industrial processes [6].

To bridge the gap between high-level intent and low-level code, specification languages were proposed. SPESC [16], for example, allows for defining contract specifications with temporal and logical expressions but remains a theoretical model without real-world vulnerability detection capabilities. A more integrated approach is found in the Move language [40], which features a built-in formal verifier, the Move Prover [22]. It uses Floyd-Hoare logic to formally verify code against specifications, including preconditions, postconditions, and invariants. While this provides a high degree of assurance, it requires developers to write formal specifications, a non-trivial task, and its design choices have different implications for verification compared to languages like Solidity [23]. A broader overview of formal methods can be found in recent reviews [19].

Our work, SSB, builds on these concepts but differentiates itself in several key ways. Like Securify and VULTRON, SSB is a semantic-based tool. However, instead of general-purpose or financial invariants, SSB focuses on a **five-dimensional invariant framework** (security, functionality, consistency, time, resource) specifically modeled for the physical and logical constraints of ICS [4]. Unlike formal verification tools like the Move Prover, SSB employs a more accessible *dynamic verification* approach within a sandbox environment, testing the contract against specifications at runtime. Furthermore, SSB leverages LLMs to assist in the automatic generation of specifications from source code and documentation, a burgeoning field of research [26,27,28], thereby lowering the barrier to entry for creating comprehensive test suites. This unique combination of ICS-specific semantic modeling, dynamic verification, and LLM-assisted specification generation allows SSB to detect subtle semantic bugs that other tools might miss, such as the premature operation of equipment due to misunderstood temporal dependencies.

## 3. Methods

### 3.1. Industrial Control Invariants Based on Contract Semantics

**Figure 1:**
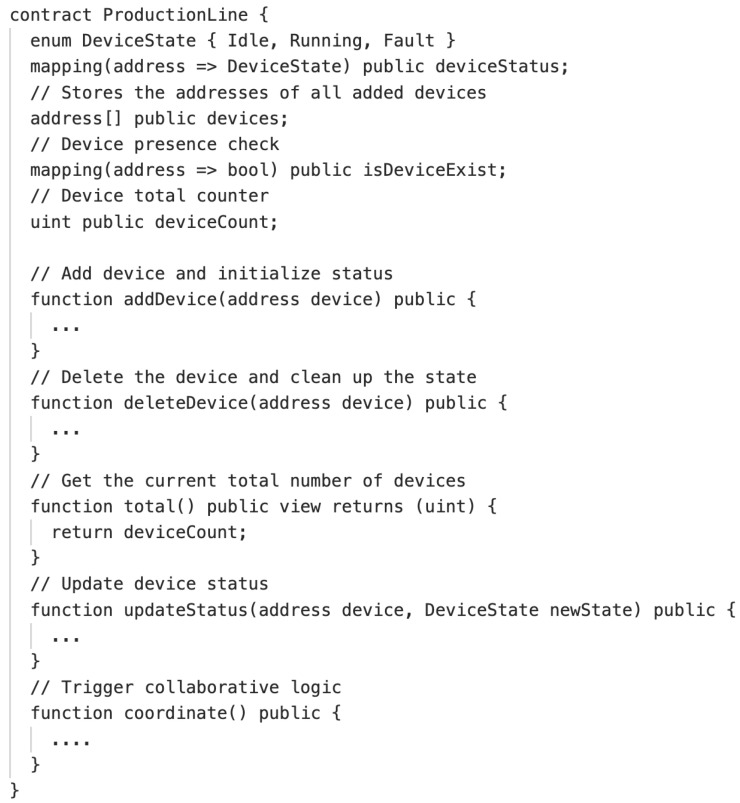
ProductionLine contract.

Current research on smart contract invariants in industrial control scenarios remains insufficient, lacking a comprehensive invariant modeling framework. This deficiency often results in overly restrictive or overly lenient invariant constraints during semantic-based security detection, thereby affecting the accuracy of specifications in evaluating smart contracts. To address this issue, this article presents a specific example of semantic security analysis for smart contracts in industrial control scenarios (as shown in Figure 1). While a full exploration is beyond this paper’s scope, our proof-of-concept ontology defines key ICS entities (e.g., devices, states, operations, temporal constraints) and their relationships. This model directly informs the five-dimensional invariants used in our framework, enabling the mapping of physical control semantics to contract code.

In this paper, a smart contract is defined as SC(dep,owner,us), where SC represents the smart contract, dep is the deployer address of the contract (typically also the owner), owner is the actual owner address of the contract, and us is the set of all user addresses associated with the contract ($u_{1},u_{2},\ldots ,u_{n}$). Additional information about users and the contract can be obtained through the operation interfaces defined in Section 3.2.1. In the context of invariant detection for smart contract specifications, we categorize common invariants into several types, covering requirements related to security, functionality, consistency, and other aspects.

**Figure 2:**
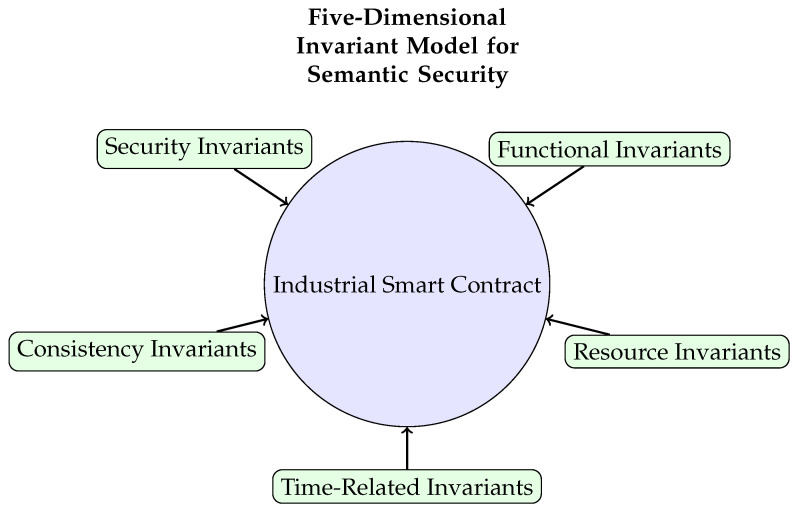
Conceptual diagram of the five-dimensional invariant modeling framework for ensuring semantic integrity in ICS smart contracts.

We model invariants based on a five-dimensional framework to ensure the precision of specification definitions shown in Figure 2. A smart contract is defined as SC(dep,owner,us).

#### 3.1.1. Security Invariants

Security invariants ensure that a contract remains in a secure state throughout its execution. These invariants are typically designed to prevent common attacks and vulnerabilities, such as reentry attacks, overflows, and privilege escalation. For instance, a contract might require that a user’s balance never becomes negative after an operation or that the caller’s permissions are validated before executing a specific function. Taking unauthorized access as an example, the updateStatus function in the ProductionLine contract allows updating the device status, but this function can only be invoked by the contract owner or authorized users. If an unauthorized user attempts to update the device status, the transaction will be reverted. This leads to the following invariants:

(1) $$(u_{1}\to updateStatus\left(device,newState\right))==false$$

(2) (owner→updateStatus(device,newState))==true

where:

- updateStatus(device,newState) represents invoking the updateStatus function to change the status of the device at address device to newState.
- $u_{1}$ represents any unauthorized user.
- The invariant (1) indicates that an unauthorized user $u_{1}$ attempting to invoke updateStatus to modify the device status should fail.
- The invariant (2) indicates that the contract owner (or an authorized user) invoking updateStatus to modify the device status should succeed.

#### 3.1.2. Functional Invariants

Functional invariants ensure that the logic of a smart contract consistently meets the expected behavior. These invariants are used to verify whether the contract’s functions operate as intended and prevent operations from producing unintended side effects. Using the updateStatus function of the ProductionLine contract as an example, its logical purpose is to correctly update the device status, as expressed by:

(3) post(deviceStatus[device])==newState

where:

- post(·) represents the state after the transaction is executed.
- newState represents the updated device status provided by the user.
- device represents the device being modified.
- deviceStatus represents the mapping in the contract that stores the collection of device statuses.
- The invariant (3) indicates that after executing the updateStatus function, the device status should match newState.

#### 3.1.3. Consistency Invariants

Consistency invariants ensure that the data within a contract remains consistent at all times and is not rendered inconsistent by certain operations. These invariants are crucial for maintaining dependencies between multiple operations and the stability of the contract state. For example, in the ProductionLine contract, the total number of devices should remain constant unless the contract owner invokes a function to increase or decrease the number of devices. Other user transactions should not alter the total number of devices, leading to the following invariant:

(4) total(devices)==deviceCount

where:

- total(devices) represents the total number of devices.
- deviceCount represents the variable in the smart contract that records the total number of devices.

#### 3.1.4. Time-Related Invariants

Time-related invariants are used in scenarios where contracts must adhere to specific temporal constraints. For instance, the contract code might only check the instantaneous state of a robotic arm (deviceStatus[armAddress] == Running) without verifying whether it has been running for a sufficient duration (e.g., a required runtime of “5 s”). This could result in the conveyor belt starting prematurely before the robotic arm completes its material handling task, potentially causing material collisions. This leads to the following invariants:

(5) (u→coordinate())==true,(timestamp−startTime≥5)

(6) (u→coordinate())==false,(timestamp−startTime<5)

where:

- coordinate() represents the function that triggers coordination logic.
- *u* represents the individual invoking the function.
- timestamp represents the current blockchain timestamp.
- startTime represents the start time of the robotic arm’s operation.
- The invariant (5) indicates that starting the conveyor belt after the robotic arm has been running for at least five seconds should succeed.
- The invariant (6) indicates that starting the conveyor belt before the robotic arm has run for five seconds should fail.

#### 3.1.5. Resource Consumption Invariants

Resource consumption invariants ensure that a contract does not exceed predefined resource limits during execution. These invariants are designed to prevent contract execution failures due to excessive gas or other resource consumption, ensuring the system’s efficiency and sustainability. The general form of such invariants for specifications can be expressed as:

(7) gas(transaction)≤targetGas

where:

- gas(transaction) represents the gas cost incurred by executing the transaction.
- targetGas represents a threshold value that should exceed the gas cost under normal circumstances, typically set to twice the normal gas consumption for the transaction.

This section provides critical theoretical support for constructing a comprehensive and scalable specification framework for smart contracts in industrial control scenarios by analyzing invariants. This framework ensures the correctness and security of contract specifications across multiple dimensions.

### 3.2. Semantic-Based Security Detection Tool for Smart Contracts

Through Section 3.1, which establishes semantic invariants for smart contract code based on industrial control scenarios, this paper proposes a semantic security detection tool for smart contracts tailored to industrial control scenarios, named SSB (Simulation Sandbox). SSB operates based on semantic reconstruction of interface operations and integrates runtime monitoring with invariant detection techniques to design a contract vulnerability detection method grounded in semantic constraints.

The architecture of the SSB tool is composed of two main stages: specification generation and dynamic verification. First, we employ a large language model (LLM) to automatically generate formal specifications from smart contract artifacts. Subsequently, these specifications are dynamically verified against the contract’s execution within a secure sandbox environment. Our implementation utilizes a custom in-memory EVM that provides full state snapshot and revert capabilities, ensuring transactional isolation between test cases. The detailed procedures are presented in Algorithms 1–3.

Algorithm 1 details the process of generating security and functional invariants from the contract’s bytecode, application binary interface (ABI), and functional documentation. This leverages the contextual understanding capabilities of a large language model (LLM) to create a comprehensive set of formal specifications. Specifically, we utilized LLM, including GPT-3.5-Turbo, GPT-4o and GPT-4o-Mini, for this task. The prompt engineering process, encapsulated in the ConstructPrompt function, structures the contract’s source code, ABI, and functional descriptions to effectively query the model for relevant invariants based on our five-dimensional framework.

**Algorithm 1** Specification generation algorithm based on LLM**Require:** Bytecode *B*, Application Binary Interface *A*, Functional Documentation *D***Ensure:** Formal Specification set Spec
  1:

Spec←∅

  2:

SourceCode←Decompile(B)

  3:

Prompt←ConstructPrompt(SourceCode,A,D)

  4:

Response←LLM.query(Prompt)

  5:

InvariantList←ParseResponse(Response)

  6:**for all** inv in InvariantList **do**  7:       **if** ValidateFormat(inv) **then**  8:             Spec←Spec∪{inv}  9:       **end if**10:
**end for**
11:**return** Spec


Once the specifications (Spec) are generated, the SSB tool proceeds to the dynamic verification stage, as outlined in Algorithm 2. This involves deploying the smart contract in an isolated sandbox, generating and executing a series of transactions designed to test each invariant, and recording the execution trace.
**Algorithm 2** Sandboxed execution algorithm for smart contracts**Require:** Bytecode *B*, Specification set Spec**Ensure:** Verification Result Result  1:E←InitializeSandbox()  2:C←DeployContract(B,E)  3:TxList←∅  4:**for all** inv in Spec **do**  5:       tx←GenerateTransactionFor(inv)  6:       TxList.add(tx)  7:**end for**  8:Trace←E.execute(TxList)  9:Violations←∅10:**for all** inv in Spec **do**11:      **if** CheckViolation(Trace, inv) **then**12:            Violations.add(inv)13:      **end if**14:**end for**15:Result←FormatResult(Violations)16:**return** Result

The core of the verification logic lies in checking the execution trace against each invariant. Algorithm 3 provides a detailed view of this validation process. It parses each invariant into its constituent parts—pre-condition, action, and post-condition—and systematically searches the execution trace for any state transitions that violate the specified logic.
**Algorithm 3** Invariant validation algorithm based on execution trace**Require:** Execution Trace *T*, Invariant *I***Ensure:** Boolean IsViolated  1:(Pre,Act,Post)←ParseInvariant(I)  2:MatchingStates←FindStates(T,Pre)  3:**for all** $S_{pre}$ in MatchingStates **do**  4:      $S_{post}\leftarrow \mathrm{FindNextState}\left(T,S_{pre},Act\right)$  5:      **if** Not Matches($S_{post}$, Post) **then**  6:             **return true**    // Violation found  7:      **end if**  8:**end for**  9:**return false**    // No violation

#### 3.2.1. Operation Definitions

To facilitate user simulation of real-world industrial control scenarios, this paper designs the following operations for SSB:

- **depSC:** Deploys a smart contract by compiling source code on the file system and using initialization bytecode for deployment.
- **setUser:** Sets the Ether balance for a specific user or creates a new user and assigns a balance to them.
- **callFunction:** Initializes and executes a transaction based on the provided function ABI and parameters.
- **expression:** Executes a JavaScript code snippet and extracts the value of a JavaScript variable into a defined variable.
- **check:** Executes a JavaScript expression and checks if it returns True. If the return value is not True, the SSB execution for the test case will terminate.
- **builtIn:** Invokes a built-in JavaScript module.
- **setEnv:** Sets blockchain environment variables, such as block number and timestamp.
- **getEnv:** Retrieves blockchain environment variables, such as block number and timestamp, and stores them in defined variables.

#### 3.2.2. Task Creation

The security detection process of SSB for smart contracts revolves around the creation and execution of tasks, which are the core components of SSB’s security evaluation mechanism.

Algorithm 4 creates a ProductionLine task aimed at conducting specification detection for the ProductionLine smart contract. This process is elaborated below using this specification detection task as an example.
**Algorithm 4** ProductionLine task  1:**Require:** Define the source code path of the smart contract: path  2:**Ensure:** All the results of the implementation of the rules: λ  3:SCAddr,dep←depSC(path)                                                                                          ▷ Deploy the smart contract  4:u1Addr←setUser()                                           ▷ Call the "setUser" operation to create a user on the blockchain  5:u2Addr←setUser()  6:d1Addr←setUser()                                        ▷ Call the "setUser" operation to create a device on the blockchain  7:d2Addr←setUser()  8:callFunction(SCAddr,addDevice(d1Addr))                                                          ▷ Add devices to the smart contract  9:callFunction(SCAddr,addDevice(d2Addr))10:callFunction(SCAddr,updateStatus(d1Addr,Idle))                 ▷ Initialize the status of the devices on the blockchain11:callFunction(SCAddr,updateStatus(d2Addr,Running))12:Define the output sets resulting from the execution of rule1: Ω13:Ω←Rule1(u1Addr,d1Addr,SCAddr)                                                                                    ▷ Execute rules detection14:Define the output sets resulting from the execution of rule2: ω15:ω←Rule2(u1Addr,d1Addr,d2Addr,SCAddr)16:**return** All the results of the implementation of the rules: λ←ω∪Ω

- **Variable definition:** Unlike specifications, task creation involves only one type of variable: global variables. As the initial step in SSB specification detection, task creation primarily aims to establish a universal initial on-chain state for specification detection and select applicable rules and their inputs. This process is executed only once during SSB detection, eliminating the need for other variable types.
- **Environment setup:** The primary objective of environment setup is to provide an initial on-chain state for specifications, including operations such as contract deployment, user creation, and device addition. Lines 1–9 in Algorithm 4 illustrate this process. First, two users and devices are created on the blockchain using SSB’s setUser operation. Then, the addDevice() function of the smart contract is invoked to add devices to the contract. Subsequently, the updateStatus() function is called to set the device statuses, preparing the environment for the execution of relevant rules.
- **Specification detection:** At this stage, suitable rules are selected for the smart contract under evaluation. SSB provides methods for creating rules tailored to smart contracts.

#### 3.2.3. Specification Framework

**Figure 3:**
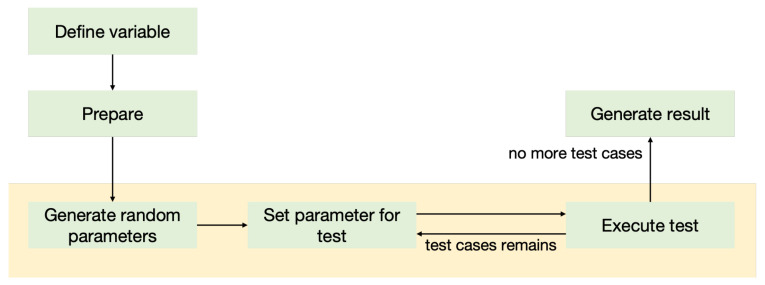
SSB rule execution workflow.

The SSB detection process relies on operational specifications for industrial control. By extracting these operational rules, this paper constructs a semantic-based specification framework. As shown in Figure 3, the framework illustrates the SSB rule execution workflow, comprising four stages: variable definition, preparation, random value generation, and execution.

Algorithm 5 presents a specification for an industrial control scenario—ensuring that the conveyor belt runs for five seconds when executing coordinate() (as discussed in Section 3.1.4).
**Algorithm 5** The robotic arm needs to run for five seconds when calling coordinate()**Require:** Smart contract deployment address: SCAddr, Smart Contract Owner: owner, Address of device: d1Addr**Ensure:** All cases λ  1:startTime←getEnv(timestamp)                                                                   ▷ Get the timestamp of d1Addr runtime  2:startHeight←getEnv(height)                                                                    ▷ Get the block height of d1Addr runtime  3:callFunction(SCAddr,updateStatus(d1Addr,Running))  4:ξ←builtIn(0<time<5)                                                       ▷ Generate a random set of parameters on request  5:Failedcasesω←∅  6:SucceedcasesΩ←∅  7:**for** time∈ξ **do**                                                                                                                           ▷ Create a new case  8:      finishTime←expression(startTime+time)  9:      finishHeight←expression(startHeight+time/2)10:      setEnv(timestamp,finishTime)                                                                                                ▷ Set the timestamp11:      setEnv(height,finishHeight)                                                                                                             ▷ Set the block12:      result←callFunction(SCAddr,coordinate())13:      **if** result == false **then**14:           Ω←Case∪Ω                                                                                     ▷ Add Case to the success collection15:      **else**16:           ω←Case∪ω                                                                                         ▷ Add Case to the failed collection17:      **end if**18:**end for**19:**return** All cases λ←Ω∪ω

- **Variable definition stage:** During the variable definition stage, variables used in the specification are categorized into four types: input variables, global variables, random variables, and execution variables (all variables used in Algorithm 5 must be predefined).
  - **Input variables:** Initialized by the user during task creation and received by the specification file (SCAddr, owner).
  - **Global variables:** Obtained during the preparation stage, such as key state information before invoking a function (e.g., startTime, startHeight).
  - **Random variables:** Random values generated by SSB (time), used to simulate various execution scenarios.
  - **Execution variables:** Parameters returned by the system after transaction execution, such as blockchain state post-function call (e.g., finishTime, finishHeight).
- **Preparation stage:** The focus of the preparation stage is to construct and modify the state of the blockchain sandbox environment. This includes, but is not limited to, obtaining block height, timestamp, and user balance. To enhance the usability and flexibility of specifications, SSB designs corresponding operations to be invoked during this stage. These operations allow users to call functions in smart contracts and retrieve or modify on-chain states at appropriate times. Lines 1–3 in Algorithm 5 illustrate this stage, where block height and timestamp are obtained through the getEnv() operation. This stage is executed only once during the specification process to avoid resource waste from repeated executions.
- **Random value generation stage:** In this stage, a set of random numbers is generated by executing built-in operations and invoking JavaScript modules. Specifications can predefine the range for random number generation and use specific algorithms to finely control the generated random values, simulating extreme values, minimal values, or other potential anomalies. Through this random number generation process, not only can contract behavior be optimized, but potential errors or malicious attack risks can also be effectively mitigated, enhancing the robustness and security of contracts across various scenarios.
- **Execution stage:** The execution stage is the core of the entire specification execution process, encompassing the simulation of smart contract transactions and the capture and validation of on-chain states. During this stage, the contract is rigorously simulated and executed, with continuous monitoring of state changes before and after transactions to ensure they align with expected outcomes. Through precise comparison of transaction states, SSB can identify potential security vulnerabilities or abnormal behaviors in smart contracts, assisting developers in making corrections and optimizations before deployment. SSB executes multiple iterations based on the generated random number set during the specification execution, reverting to the previous on-chain state after each iteration to generate different test cases. Lines 7–18 in Algorithm 5 demonstrate this stage: first, the expression operation calculates the end time and block height; then, setEnv sets the blockchain state and invokes the coordinate() function; finally, SSB evaluates whether the test cases meet the expected outcomes based on the predefined invariants in the specification.

## 4. Results

### 4.1. Evaluation Metrics

In evaluating the effectiveness of automatically generating smart contract specifications, it is crucial to employ metrics that assess both the functional correctness and the structural integrity of the output. To this end, this study utilizes a combination of **Pass@k** to measure functional success and **Jaccard Similarity** based on a graph representation to evaluate structural resemblance.

The Pass@k metric is widely used to evaluate the performance of code generation models. It measures the probability that at least one of a batch of *k* generated candidate samples for a given problem will pass a set of predefined tests (e.g., unit tests). This metric is particularly useful for assessing whether a model is capable of producing a functionally correct solution within a given number of attempts. The unbiased estimator for Pass@k is calculated as follows:

(8) Pass@k=Eproblems1−n−cknk

where *n* is the total number of generated samples, and *c* is the number of correct samples among them. However, Pass@k has limitations; it treats all failing samples equally, even though some may be structurally very close to a correct solution and thus provide valuable insights for refinement.

To overcome the limitations of Pass@k and provide a more nuanced evaluation, this study introduces an analysis based on graph similarity. In this context, an incorrectly generated specification that fails the Pass@k check can be considered a False Positive, while a ground-truth invariant the LLM fails to generate represents a False Negative. Each smart contract specification is converted into a directed graph where nodes represent variables and operations, and edges represent the flow of data between them (i.e., inputs and outputs).

By representing specifications as graphs, we can compare their structural similarity using the Jaccard Similarity Index. This index measures the similarity between two finite sets and is defined as the size of their intersection divided by the size of their union. We calculate the Jaccard similarity for three distinct sets derived from the graphs: the set of variable nodes (*V*), the set of operation nodes (*O*), and the set of edges (*E*). For two graphs, $G_{1}$ and $G_{2}$, the similarities are calculated as:

(9) $$J_{\mathrm{vars}}\left(G_{1},G_{2}\right)=\frac{|V_{1}\cap V_{2}|}{|V_{1}\cup V_{2}|}$$

(10) $$J_{\mathrm{ops}}\left(G_{1},G_{2}\right)=\frac{|O_{1}\cap O_{2}|}{|O_{1}\cup O_{2}|}$$

(11) $$J_{\mathrm{edges}}\left(G_{1},G_{2}\right)=\frac{|E_{1}\cap E_{2}|}{|E_{1}\cup E_{2}|}$$

This method allows for a detailed assessment of how closely a generated specification matches a ground-truth reference in terms of its core components and logic flow, offering a more comprehensive measure of quality beyond simple pass/fail functional tests.

### 4.2. Experimental Design and Dataset

#### 4.2.1. Experimental Subjects and Environmental Constraints

We agree that validation in a live industrial environment would be the ideal proof of our framework’s practical applicability. However, **obtaining access to real-world industrial control systems for security testing is exceptionally difficult due to stringent confidentiality policies and the significant operational risks involved**. To mitigate this limitation while ensuring a rigorous evaluation, we adopted a standard and robust methodology widely used in security research: we constructed a comprehensive dataset of smart contracts and **injected them with a wide array of known vulnerabilities**. This approach allows for a controlled, reproducible, and thorough assessment of our tool’s detection capabilities against a ground-truth benchmark.

For our validation subjects, we selected widely adopted open-source smart contracts, including those based on the ERC20 standard. As comparison benchmarks, we chose three influential semantic analysis tools: ZEUS [39], Securify [33], and VULTRON [34], which represent diverse technological approaches in the field.

#### 4.2.2. Smart Contract Sample Set

To comprehensively assess SSB’s performance in vulnerability detection, this study constructs a sample set comprising 58 smart contracts. These contracts cover mainstream token standards, including but not limited to ERC20 and ERC721, aiming to reflect the diversity present in real-world ecosystems. To systematically test the vulnerability detection capabilities of the tools, a total of 258 common security vulnerability instances were manually injected into the sample contracts. The types of injected vulnerabilities include:

- Common security vulnerabilities: such as integer overflow/underflow (IO/IU), reentrancy (RE), access control (AC), exception handling errors (EHE), denial of service (DoS), type confusion (TC), unexpected function call (UFC), and ether frozen (EF).
- Logical vulnerabilities: simulating errors that developers might introduce due to coding oversights or misunderstandings of logic.

This injected dataset provides a standardized evaluation benchmark for subsequent specification detection experiments.

#### 4.2.3. Safety Specification Set

Targeting the core security requirements and common vulnerability patterns of token standards such as ERC20, this study precisely defines and formalizes 192 safety specifications. These specifications cover security constraints for critical operations, including token transfer, authorization, balance management, access control, and exception handling. All specifications are rigorously implemented based on the SSB specification framework, ensuring that they can be effectively parsed and verified by the SSB tool.

### 4.3. Experimental Results and Analysis

#### 4.3.1. Comparison of Vulnerability Type Coverage

Table 2 presents the comparison results of vulnerability detection coverage across eight critical vulnerability types for SSB and the baseline tools. The experiments demonstrate that SSB exhibits the most comprehensive vulnerability coverage, successfully detecting all eight vulnerability types covered in this study. In contrast:

- ZEUS performs well in detecting reentrancy and integer overflow vulnerabilities but has noticeable blind spots in other vulnerability types such as access control and exception handling.
- Securify is capable of detecting reentrancy, exception handling, type confusion, unexpected function call, and ether frozen vulnerabilities, but it lacks sufficient detection capabilities for integer overflow and access control vulnerabilities.
- VULTRON can detect reentrancy, integer overflow, and exception handling vulnerabilities, but it fails to cover other types such as access control, denial of service, and type confusion.

These results clearly indicate that SSB significantly outperforms the selected baseline tools in terms of the breadth of vulnerability type coverage.

#### 4.3.2. Quantitative Analysis of Vulnerability Detection Capabilities

**Figure 4:**
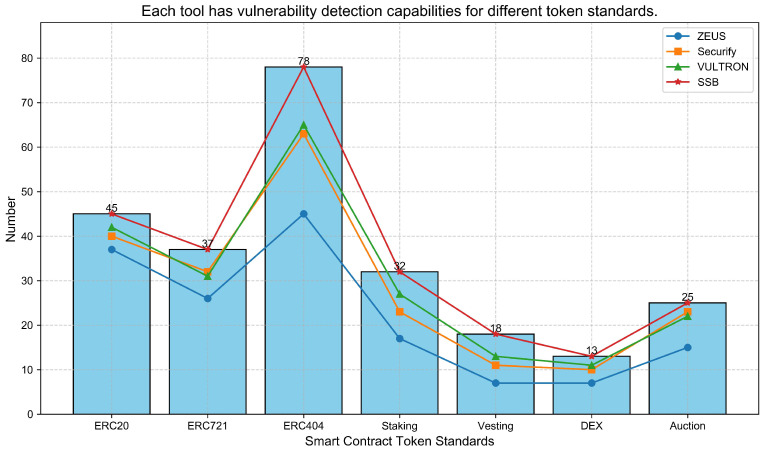
Comparison of vulnerability detection capabilities of smart contract security tools (bar chart—total preset vulnerabilities; line chart—actual detected count by each tool).

Figure 4 visually illustrates the overall detection performance of each tool across a set of 58 smart contract samples with manually injected vulnerabilities. In the figure:

- The height of the bars represents the total number of preset vulnerabilities of various types in the sample set (a total of 258 vulnerabilities).
- The points on the line graph represent the number of vulnerabilities actually detected by each tool.

The experimental results show that SSB performs exceptionally well in this evaluation. As illustrated in Figure 3, the number of vulnerabilities detected by SSB exactly matches the total number of preset vulnerabilities, achieving a 100% detection rate for the specific 258 vulnerabilities injected into our test set.

It is crucial, however, to qualify this finding. The 100% detection rate was achieved for vulnerabilities that were explicitly covered by our manually crafted specification set. This perfect score serves as a strong validation of the soundness and effectiveness of SSB’s dynamic verification engine when it is provided with a correct and relevant specification.

Therefore, the overall performance of the SSB framework is fundamentally dependent on the completeness and quality of the specification set. If a vulnerability type is not covered by a specification, SSB will not detect it. This limitation highlights the risk of overfitting to the known specifications and underscores that the 100% result is not a claim of detecting all possible vulnerabilities in any contract. Rather, it confirms that SSB exhibits significantly superior and reliable detection capabilities for specified properties within the controlled experimental settings of this study.

>Regarding system performance and applicability in real-time scenarios, it is important to distinguish between the two stages of our framework. The **LLM-based specification generation is a one-time, offline process** performed prior to deployment. Its duration does not affect the runtime performance of the system being secured. The **dynamic verification occurs within the sandboxed environment and is highly efficient**, as it involves executing targeted transactions and checking states against pre-compiled invariants. This makes our approach well-suited for pre-deployment auditing and continuous integration/continuous deployment (CI/CD) pipelines, which are the primary use cases for such security analysis tools.

## 5. Conclusions

This paper introduces SSB, a novel semantic security framework for smart contracts in industrial control scenarios. By establishing a five-dimensional invariant model, our framework achieves a more precise semantic mapping from industrial control logic to contract code, addressing critical vulnerabilities that traditional tools may miss.

### Limitations and Future Directions

We acknowledge the limitations of this study, which open important avenues for future research. The primary limitation is the **lack of validation in a real-world ICS environment**, as discussed in Section 4.2.1. While our use of a benchmark with injected vulnerabilities provides a controlled and rigorous assessment, the ultimate test of applicability will require deployment in a live or high-fidelity simulated setting.

Furthermore, our current work focuses specifically on the semantic security of the smart contract logic layer. While our vulnerability injection methodology is threat-driven, we acknowledge that a formal threat modeling process could be a valuable complementary activity to guide specification generation. Broader IIoT challenges, such as network-level packet loss, physical clock synchronization, and resilience to specific cyberattacks on the underlying infrastructure, are not within the scope of this paper. These factors are critical for end-to-end security.

To address these points, we propose the following directions for future work:

1. **Integration with digital twins:** To bridge the gap between on-chain logic and physical-layer realities, we plan to integrate SSB with industrial digital twin technologies. This would enable a joint validation framework that can simulate the effects of contract execution on physical equipment and test resilience against challenges like time synchronization errors between the blockchain and PLCs. Future work will also focus on aligning our underlying ontology with industry standards like IEC 62443 to ensure broader applicability.
2. **Cross-chain interoperability:** To adapt to multi-chain architectures in complex industrial ecosystems (e.g., involving Polkadot or Cosmos), future work will explore mechanisms for ensuring semantic consistency across heterogeneous blockchains.
3. **Adaptive specification generation:** To improve the completeness of our specification set, we will continue to refine our LLM-based approach, developing adaptive models that can automatically synthesize more complex invariants through deeper control flow analysis and vulnerability pattern matching.
4. **Specification lifecycle management:** To address the challenge of evolving smart contracts and potential "specification drift," future research will investigate methods for maintaining and updating security specifications over the long-term lifecycle of an ICS, ensuring security is preserved across contract upgrades.
5. **Developer usability studies:** To translate our research prototype into a practical, developer-friendly tool, future work must include user studies to evaluate the usability of the SSB framework, including the process of reading outputs and refining specifications.

## Figures and Tables

**Table 1:** Comparison of mainstream smart contract security detection methods.

Category	Approach	Examples
**Static analysis**	Scans code for vulnerability patterns (e.g., reentrancy, overflow). Fast, but may miss issues or flag false positives.	Oyente [29], Slither [20], SmartCheck [30]
**Dynamic analysis**	Tests contract with inputs (fuzzing, symbolic execution) to find vulnerabilities.	Mythril [21], Manticore [31], Echidna [32]
**Formal verification**	Proves contract meets formal specs using theorem provers. High assurance, but complex.	Move Prover [22], Certora, K-Framework [24]
**Semantic-based analysis**	Detects violations of logical properties or code intent.	Securify [33], VULTRON [34]
**AI/LLM-based**	Uses ML/LLMs to find complex vulnerabilities in code.	GPTScan [26], SmartLLMSentry [28]

**Table 2:** Comparison of vulnerability coverage between SSB and existing specification detection tools.

Detection Tool	Detectable Vulnerability Types							
	**RE**	**IO/IU**	**AC**	**EHE**	**DOS**	**TC**	**UFC**	**EF**
ZEUS	✔	✔						
Securify	✔			✔		✔	✔	✔
VULTRON	✔	✔		✔				
SSB	✔	✔	✔	✔	✔	✔	✔	✔

Note: ✔ indicates that the tool can effectively detect the corresponding type of vulnerability.

## Data Availability

The datasets presented in this article are not readily available because confidentiality principle. Requests to access the datasets should be directed to 22210240162@m.fudan.edu.cn.

## References

[1] Stouffer K., Falco J., Scarfone K. (2011). Guide to industrial control systems (ICS) security. Nist Spec. Publ..

[2] Ralston P.A.S., Graham J.H., Hieb J.L. (2007). Cyber security risk assessment for SCADA and DCS networks. ISA Trans..

[3] Zheng Z., Xie S., Dai H.-N., Chen X., Wang H. (2018). Blockchain challenges and opportunities: A survey. Int. J. Web Grid Serv..

[4] Essaid M., Ju H. (2025). Blockchain Solutions for Enhancing Security and Privacy in Industrial IoT. Appl. Sci..

[5] Chang Y.H., Peng T.E., Li J.S., Liu I.H. Industrial Control System State Monitor Using Blockchain Technology. Proceedings of the 29th International Conference on Artificial Life and Robotics.

[6] Zhang J., Zhong S., Wang J., Wang L., Yang Y., Wei B., Zhou G. A review on blockchain-based systems and applications. Proceedings of the Internet of Vehicles. Technologies and Services Toward Smart Cities.

[7] Zou W., Lo D., Kochhar P.S., Le X.-B.D., Xia X., Feng Y., Chen Z., Xu B. (2019). Smart contract development: Challenges and opportunities. IEEE Trans. Softw. Eng..

[8] Lai E., Luo W. Static analysis of integer overflow of smart contracts in Ethereum. Proceedings of the 2020 4th International Conference on Cryptography, Security and Privacy.

[9] Iuliano G., Di Nucci D. (2024). Smart Contract Vulnerabilities, Tools, and Benchmarks: An Updated Systematic Literature Review. arXiv.

[10] Langmann R., Rojas-Peña L.F. A PLC as an Industry 4.0 component. Proceedings of the 2016 13th International Conference on Remote Engineering and Virtual Instrumentation (REV).

[11] Boyes H., Hallaq B., Cunningham J., Watson T. (2018). The industrial internet of things (IIoT): An analysis framework. Comput. Ind..

[12] Madakam S., Ramaswamy R., Tripathi S. (2015). Internet of Things (IoT): A literature review. J. Comput. Commun..

[13] Wood G. (2016). Polkadot: Vision for a heterogeneous multi-chain framework. White Pap..

[14] Kwon J., Buchman E. (2019). Cosmos whitepaper. A Netw. Distrib. Ledgers.

[15] Hasan M. (2024). A Study on the Integration of Blockchain Technology for Enhancing Data Integrity in Cyber Defense Systems. J. Digit. Transform. Cyber Resil. Infrastruct. Secur..

[16] He X., Qin B., Zhu Y., Chen X., Liu Y. SPESC: A specification language for smart contracts. Proceedings of the 2018 IEEE 42nd Annual Computer Software and Applications Conference (COMPSAC).

[17] Belchior R., Guerreiro S., Vasconcelos A., Correia M. (2022). A survey on business process view integration: Past, present and future applications to blockchain. Bus. Process Manag. J..

[18] Zhu H., Yang L., Wang L., Sheng V.S. (2024). A Survey on Security Analysis Methods of Smart Contracts. IEEE Trans. Serv. Comput..

[19] Kiani R., Sheng V.S. (2024). Automated Repair of Smart Contract Vulnerabilities: A Systematic Literature Review. Electronics.

[20] Feist J., Grieco G., Groce A. Slither: A static analysis framework for smart contracts. Proceedings of the 2019 IEEE/ACM 2nd International Workshop on Emerging Trends in Software Engineering for Blockchain.

[21] Mueller B. (2020). Introducing Mythril: A Framework for Bug Hunting on the Ethereum Blockchain. Medium. https://medium.com/hackernoon/introducing-mythril-a-framework-for-bug-hunting-on-the-ethereumblockchain-9dc5588f82f6.

[22] Zhong J.E., Cheang K., Qadeer S., Grieskamp W., Blackshear S., Park J., Zohar Y., Barrett C., Dill D.L., Lahiri S.K., Wang C. (2020). The Move Prover. Computer Aided Verification.

[23] Bartoletti M., Crafa S., Lipparini E. (2025). Formal verification in Solidity and Move: Insights from a comparative analysis. arXiv.

[24] Hildenbrandt E., Saxena M., Rodrigues N., Zhu X., Daian P., Guth D., Moore B., Park D., Zhang Y., Stefanescu A. Kevm: A complete formal semantics of the ethereum virtual machine. Proceedings of the 2018 IEEE 31st Computer Security Foundations Symposium.

[25] Krichen M., Lahami M., Al–Haija Q.A. Formal methods for the verification of smart contracts: A review. Proceedings of the 2022 15th International Conference on Security of Information and Networks.

[26] Sun Y., Wu D., Xue Y., Liu H., Wang H., Xu Z., Xie X., Liu Y. Gptscan: Detecting logic vulnerabilities in smart contracts by combining gpt with program analysis. Proceedings of the IEEE/ACM 46th International Conference on Software Engineering.

[27] Wei Z., Sun J., Zhang Z., Zhang X., Li M., Hou Z. (2024). LLM-SmartAudit: Advanced Smart Contract Vulnerability Detection. arXiv.

[28] Zaazaa O., El Bakkali H. (2024). SmartLLMSentry: A Comprehensive LLM Based Smart Contract Vulnerability Detection Framework. J. Metaverse.

[29] Luu L., Chu D.H., Olickel H., Saxena P., Hobor A. Making smart contracts smarter. Proceedings of the 2016 ACM SIGSAC Conference on Computer and Communications Security.

[30] Tikhomirov S., Voskresenskaya E., Ivanitskiy I., Takhaviev R., Marchenko E., Alexandrov Y. Smartcheck: Static analysis of ethereum smart contracts. Proceedings of the 1st International Workshop on Emerging Trends in Software Engineering for Blockchain.

[31] Mossberg M., Manzano F., Hennenfent E., Groce A., Grieco G., Feist J., Brunson T., Dinaburg A. Manticore: A user-friendly symbolic execution framework for binaries and smart contracts. Proceedings of the 2019 34th IEEE/ACM International Conference on Automated Software Engineering (ASE).

[32] Grieco G., Song W., Cygan A., Feist J., Groce A. Echidna: Effective, usable, and fast fuzzing for smart contracts. Proceedings of the 29th ACM SIGSOFT International Symposium on Software Testing and Analysis.

[33] Tsankov P., Dan A., Drachsler-Cohen D., Gervais A., Bünzli F., Vechev M. Securify: Practical security analysis of smart contracts. Proceedings of the 2018 ACM SIGSAC Conference on Computer and Communications Security.

[34] Wang H., Li Y., Lin S.-W., Ma L., Liu Y. VULTRON: Catching vulnerable smart contracts once and for all. Proceedings of the 2019 IEEE/ACM 41st International Conference on Software Engineering: New Ideas and Emerging Results (ICSE-NIER).

[35] Dhillon V., Metcalf D., Hooper M. (2017). The DAO hacked. Blockchain Enabled Applications: Understand the Blockchain Ecosystem and How to Make It Work for You.

[36] Harvey C.R., Ramachandran A., Santoro J. (2021). DeFi and the Future of Finance.

[37] Wu B., Li Q., Xu K., Li R., Liu Z. Smartretro: Blockchain-based incentives for distributed iot retrospective detection. Proceedings of the 2018 IEEE 15th International Conference on Mobile Ad Hoc and Sensor Systems.

[38] Liu C., Liu H., Cao Z., Chen Z., Chen B., Roscoe B. Reguard: Finding reentrancy bugs in smart contracts. Proceedings of the 40th International Conference on Software Engineering: Companion Proceedings.

[39] Kalra S., Goel S., Dhawan M., Sharma S. ZEUS: Analyzing Safety of Smart Contracts. Proceedings of the 25th Annual Network and Distributed System Security Symposium (NDSS).

[40] Diem. 2020. *The Move Programming Language*. https://diem.github.io/move.

